# The Neutron Structure of Urate Oxidase Resolves a Long-Standing Mechanistic Conundrum and Reveals Unexpected Changes in Protonation

**DOI:** 10.1371/journal.pone.0086651

**Published:** 2014-01-23

**Authors:** Esko Oksanen, Matthew P. Blakeley, Mohamed El-Hajji, Ulf Ryde, Monika Budayova-Spano

**Affiliations:** 1 Institut de Biologie Structurale (IBS), Direction des Sciences du Vivant, Commissariat à l’Energie Atomique et aux Energies Alternatives, Grenoble, France, IBS, Centre National de la Recherche Scientifique, Grenoble, France, IBS, Université Grenoble Alpes, Grenoble, France; 2 Institut Laue-Langevin, Grenoble, France; 3 Sanofi, Montpellier, France; 4 Department of Theoretical Chemistry Lund University, Lund, Sweden; University of Oulu, Finland

## Abstract

Urate oxidase transforms uric acid to 5-hydroxyisourate without the help of cofactors, but the catalytic mechanism has remained enigmatic, as the protonation state of the substrate could not be reliably deduced. We have determined the neutron structure of urate oxidase, providing unique information on the proton positions. A neutron crystal structure inhibited by a chloride anion at 2.3 Å resolution shows that the substrate is in fact 8-hydroxyxanthine, the enol tautomer of urate. We have also determined the neutron structure of the complex with the inhibitor 8-azaxanthine at 1.9 Å resolution, showing the protonation states of the K10–T57–H256 catalytic triad. Together with X-ray data and quantum chemical calculations, these structures allow us to identify the site of the initial substrate protonation and elucidate why the enzyme is inhibited by a chloride anion.

## Introduction

**Figure 1:**
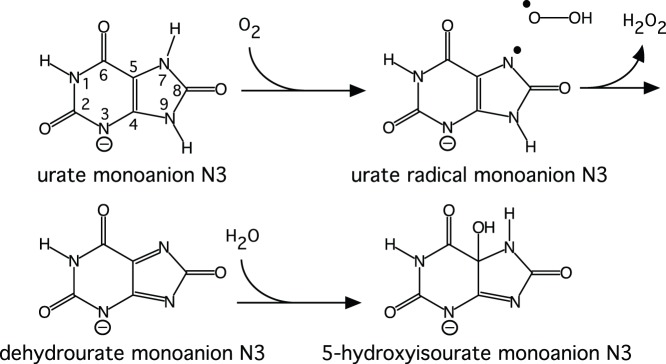
The oxidation of the monoanion N3 of uric acid by molecular oxygen through a dianion–oxygen complex best described as a biradical to dehydrourate followed by hydroxylation to 5-hydroxyisourate, which is metastable and further degraded to allantoin (not shown).

**Figure 2:**
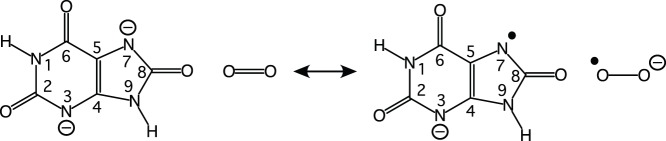
The two resonance forms of the urate dianion–oxygen pair showing the atom numbering of the urate backbone.

Urate oxidase (Uox, EC 1.7.3.3) or uricase is an enzyme involved in the metabolism of purines. It catalyses the oxidation of uric acid to metastable 5-hydroxyisourate (5-HIU) which is further degraded to allantoin (Fig. 1) either non-enzymatically [1] or with the assistance of two enzymes [2]. The oxidant is molecular oxygen, which is reduced to hydrogen peroxide. Most oxidases make use of either a transition-metal ion or an organic cofactor, but urate oxidase appears to require neither [3]. Uric acid has two p*K*_a_s of 5.4 and 9.8 [4] so at physiological pH, it is predominantly in the monoanionic form and it was shown by NMR [1] that the predominant monoanion in solution is deprotonated at N3 in accord with *ab initio* quantum chemical calculations [5]. According to these calculations, the N3,N7 dianion is most favourable for oxidation [5]. In fact, the electron transfer from the dianion to dioxygen is highly exothermic [5], so that the urate dianion–dioxygen pair is better described as a resonance hybrid (Fig. 2), with the right-hand term dominant. Therefore the key function of the enzyme is to deprotonate the monoanion; the dianion is spontaneously oxidised to the radical anion. The urate radical anion has been observed as an intermediate in the uncatalysed oxidation of urate, with the unpaired electron localised primarily on the five-membered ring of the purine structure [6].

The enzyme is a tetrameric barrel with the active site formed by residues from two monomers. In the most common crystal form the asymmetric unit contains a monomer and the tetramer is formed by the crystallographic 222 symmetry [7]. The residues R176 and Q228 bind the purine ring of the substrate and the molecular oxygen binding site above the purine plane is formed by N254 and T57*, where * denotes that the residue is from another monomer.

**Figure 3:**
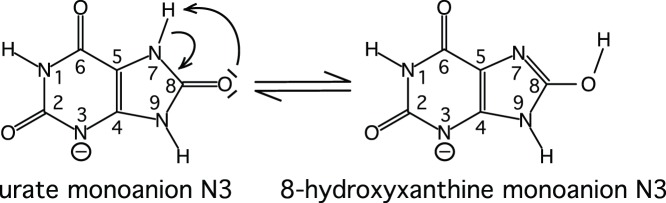
The tautomerisation of the urate monoanion N3 (keto tautomer) to the 8-hydroxyxanthine monoanion N3 (enol tautomer). The atom numbering is shown on the urate backbone.

The activity of urate oxidase is highest at pH >8 [3], but the enzyme functions at physiological pH. The pH dependence of the reaction rate V and V/K_urate_ (where K_urate_ is the Michaelis coefficient for urate) showed very similar p*K* values of ∼6.3, indicating that a protolysable group of p*K*_a_ 6.3 is involved in the catalytic step [3]. The observation that the inhibition constant *K*_i_ for 9-methyl uric acid was pH-independent while that for xanthine showed a p*K* of 7.5 [1], [8], suggested that a general base deprotonates the monoanion as the first step of catalysis. In the crystal structure, however, no obvious functional group can be identified that could deprotonate N7. In fact N7 is hydrogen-bonded to the main-chain amide of T57*, suggesting that it is already deprotonated. This is the case in complexes with inhibitors such as 8-azaxanthine, as well as in complexes with urate when inhibited by a chloride ion, which is similar in size and polarisability to the co-substrate molecular oxygen [9]. The X-ray maps only provide reliable information about the non-hydrogen atom positions and therefore could not distinguish whether the species in the active site was a urate anion, the proposed reaction intermediate dehydrourate or some other species along the reaction pathway. Similarly the X-ray maps also cannot distinguish between the tautomeric states of the urate backbone. In solution the keto tautomer (Fig. 3) is expected to be the most stable, but the enol tautomer 8-hydroxyxanthine has been suggested as a possible intermediate for a reaction at physiological pH [10]. We will refer to the arrangement of the non-hydrogen atoms as the urate backbone and the keto tautomer as urate.

Kinetic studies identified two intermediates along the reaction pathway from uric acid (presumed to be monoanionic) to 5-HIU [3]. Kahn *et al.* [11] interpreted these intermediates as the dianion and 5-hydroperoxyisourate based on the calculated optical spectra, but this interpretation has later been questioned [5]. ^18^O-labelling has shown that both oxygen atoms of the hydrogen peroxide originate from molecular oxygen [12] and the hydroxyl group in 5-HIU from the solvent [1]. However, the hydroxylation most likely occurs in the active site, as the 5-HIU was proven to be chiral [1]. It has been proposed based on computational studies that the first observed intermediate is dehydrourate [5].

Electron paramagnetic resonance (EPR) studies [13] suggested a radical mechanism where a one-electron transfer occurs from the substrate to an intermediate acceptor in the enzyme even in the absence of molecular oxygen. Under anaerobic conditions the molecular oxygen binding site is occupied by water, but the structure of the active site is otherwise very similar to structures determined under aerobic conditions [13]. Despite extensive studies, the mechanism of the key urate deprotonation step has remained elusive, and establishing the protonation states of the active-site species is crucial for a better understanding of the mechanism. The most unambiguous method to determine the proton positions is neutron crystallography [14].

With a systematic approach to crystal growth [15] we obtained very large (1–4 mm^3^) crystals [16] suitable for neutron diffraction [17]. These crystals are grown at pD 8.5 where the enzyme is most active. We present here neutron structures of urate oxidase in the presence of the inhibitor 8-azaxanthine and with the natural substrate urate, as well as the corresponding X-ray structures at atomic resolution. The structures show that the substrate is actually an 8-hydroxyxanthine monoanion. This allows us to postulate a mechanism where deprotonation occurs at O8, thus solving a long-standing mechanistic conundrum.

## Materials and Methods

### Protein Material

The hydrogenated protein was obtained from Sanofi and exchangeable hydrogens were exchanged for deuterium as described previously [16]. The crystallisation, data collection and processing were also reported previously [17]. Neutron data were collected at the LADI-III instrument at the Insitut Laue-Langevin from crystals of *A. flavus* urate oxidase grown in the presence of the substrate uric acid and the inhibitor 8-azaxanthine. It is important to note that the crystallisation conditions included 100 mM NaCl. Ambient temperature X-ray data for the joint neutron–X-ray refinement were collected with a laboratory source from crystals grown under exactly the same conditions. High-resolution X-ray data were collected at the European Synchrotron Radiation Facility to exclude artefacts from the deuterated crystallisation conditions. Urate oxidase activity was determined by monitoring the degradation of uric acid by spectrophotometry at 292 nm using a molar absorbance coefficient of 12.753 M^−1^cm^−1^. One enzyme activity unit (EAU) corresponds to the conversion of 1 µmol of uric acid per minute in a triethanolamine (TEA) buffer of pH 8.9 at 30°C.

### Crystallographic Studies

The phases for the ambient temperature X-ray data were obtained by molecular replacement in MOLREP [18] using PDB-ID 2IBA [19] as a model. Non-protein atoms were removed from the model and B-factors were set to 20 Å^2^. The models were then rigid-body refined against the X-ray data in Refmac5 [20] to Rwork/Rfree = 0.263/0.251 (8-hydroxyxanthine complex) followed by restrained refinement and some rounds of manual model building. For all data sets, 5% of the reflections were assigned to the test set. Hydrogen and deuterium atoms were added to the ambient-temperature X-ray model, which was used as a starting point for the joint X-ray–neutron refinement. The high-resolution X-ray structures were phased by molecular replacement as described above and refined separately. The resulting models were compared to the joint X-ray–neutron models and used to help the manual model building of the latter. Joint neutron–X-ray refinement (Table 1) was performed with phenix.refine in the PHENIX program suite [21], [22]. As the protein was not perdeuterated, all non-exchangeable hydrogen atoms were ^1^H. While the incoherent scattering from these hydrogens did not severely affect the neutron data collection, the negative peaks arising from the non-exchanged hydrogen atoms still complicated the nuclear-scattering density-map interpretation. Most of the water molecules visible in the maps were distinctly ‘banana shaped’, indicating that the deuterium atoms were ordered at least to some extent. However, the optimised coordinates from the joint refinement with standard settings did not always correspond to chemically reasonable hydrogen bonds. This is probably due to the limited quality of the neutron maps, in terms of both resolution and completeness. Therefore deuterium atoms that were clearly involved in hydrogen bonds were restrained to a deuterium-acceptor distance of 1.7 Å with an expected standard deviation of 0.2 Å and a donor–deuterium–acceptor angle of 180° with an expected standard deviation of 10°. Imposing these restraints had a negligible effect on the *R*-values and resulted in no significant negative peaks in the mF_o_–DF_c_ map, which indicated that the chemically more reasonable hydrogen bonding model fits the data equally well. The restraints were not used in areas where the hydrogen bond pattern was more complicated with *e.g.* bifurcated hydrogen bonds.

**Table 1:** Refinement statistics. For the joint X-ray–neutron refinements, statistics for both X-ray and neutron data are shown. All crystals had the space group I222.

	8HX complex(joint X-ray/neutron)	8HX complex(cryo X-ray)	8AZA complex(joint X-ray/neutron)	8AZA complex(cryo X-ray)
**PDB-ID**	**4N9M**	**4N9S**	**4N3M**	**4N9V**
**Unit cell ** ***a b c*** ** (Å)**	80.2 96.2 105.5	79.9 95.4 104.8	80.2 96.2 105.5	79.8 95.1 104.5
**Resolution (Å) X-ray** a	19.21–2.02 (2.10–2.02)	35.27–1.06 (1.10–1.06)	35.54–1.92 (1.9877–1.9191)	35.16–1.10 (1.14–1.10)
**Resolution (Å) neutron** a	40.1–2.30 (2.48–2.30)		52.75–1.90 (1.990–1.90)	
**No. of reflections** **X-ray/neutron**	26344 (2433)/13179(1845)	176940 (15604)	30411 (2352)/23197(1669)	158797 (15878)
**R_work_ X-ray/neutron** a,b	0.1666 (0.2286)/0.2586(0.3622)	0. 1257 (0.2066)	0.1398 (0.1870)/0.2398(0.3663)	0.1389 (0.1954)
**R_free_ X-ray/neutron** a,b	0.1887 (0.3058)/0. 2884(0.4472)	0. 1414 (0.2245)	0.1723 (0.2484)/0.2672(0.3628)	0.1532 (0.1925)
**No. of atoms (non H)**	2882	3304	2491	3057
**Protein**	2682	2808	2322	2475
**Ligand**	14	20	15	45
**Water**	186	476	169	537
**No. of H atoms**	2321	1945	2609	2219
**Protein**	1951	1942	2311	2219
**Ligand**	3	3	2	0
**Water**	367	0	294	0
**Average B-factor (Å^2^)**	19.1	16.1	24.4	15.5
**Protein**	18.1	15.0	23.8	14.3
**Ligand**	23.5	12.5	19.3	9.8
**Water**	27.8	27.1	30.9	25.9
**Bond length r.m.s.** **deviation (Å)**	0.075 c	0.007	0.094 c	0.008
**Bond angle r.m.s.** **deviation (Å)**	2.409	1.378	3.784 c	1.370

a Highest resolution shell is shown in parenthesis.

b R_work_ = ∑_hkl_(|F_o_(hkl)|–|F_c_(hkl)|)/∑_hkl_|F_o_(hkl)| for F(hkl) not belonging to the test set (5%);

R_free_ = ∑_hkl_(|F_o_(hkl)|–|F_c_(hkl)|)/∑_hkl_|F_o_(hkl)| for F(hkl) in the test set (5%).

c Including hydrogen bond restraints.

The high-resolution X-ray structures were refined (Table 1) with Refmac5 [20] and PHENIX [21], [22]. Anisotropic displacement factors were refined for all atoms and the occupancies of alternate conformations were refined per residue. In the 1.05 Å resolution urate structure, the substrate and the Cl^−^ in the active site were also treated as one occupancy group. Non-water hydrogen atom coordinates were taken from the neutron model and were refined with a ‘riding hydrogen’ model.

The map interpretation and manual model building were performed with Coot [23] for the high-resolution X-ray structures and with Coot and PyMol (The PyMOL Molecular Graphics System, Version 1.2r1, Schrödinger, LLC) for the neutron data. Figures were prepared with PyMol. The structures were deposited to the Protein Data Bank with the accession codes 4N3M, 4N9M, 4N9S and 4N9V.

### Computational Chemistry

The quantum-mechanical (QM) calculations on the isolated model systems were performed with the program Gaussian09 (Gaussian Inc.) using density functional theory (DFT) with the B3LYP functional and a 6–31++G(d,p) basis set. A water-like continuum-solvent environment was used (IEF-PCM with SMD parameters for water).

The combined QM and molecular mechanics (QM/MM) calculations were performed with the program ComQum [24], [25] using Turbomole for the quantum system and AMBER [26] for the classical MM system with the AMBER 1999 force field [27]. A tetramer of urate oxidase in complex with 8-hydroxyxanthine was first protonated and hydrated with 13819 water molecules in addition to the 824 crystallographic water molecules. The added protons and waters were then equilibrated by 45 ps simulated annealing with the program Sander in the AMBER suite, keeping the crystallographic coordinates fixed. Only one active site in the tetramer was included in the quantum system and it consisted of 117 atoms. DFT was used for all the QM calculations. A geometry optimisation was first performed using the TPSS functional and the def2-SVP [28] basis set, followed by a single-point energy calculation using the B3LYP functional and the def2-TZVP [29] basis set. The rest of the structure was initially kept fixed during the QM geometry optimisation. Releasing the classical part did not lead to significant changes in coordinates.

## Results and Discussion

We have determined the neutron structure of recombinant *A. flavus* urate oxidase in complex with 8-azaxanthine at 1.9 Å resolution and with the substrate urate at 2.3 Å resolution. A joint neutron–X-ray refinement (Table 1) was performed using an X-ray data set collected at the same temperature from crystals grown under exactly the same conditions. As the neutron experiments require that the crystals are grown or soaked in D_2_O, we have also determined the X-ray structure of the complexes in D_2_O at 1.1 Å and 1.06 Å resolutions, respectively, at 100 K, and these show no major differences from the urate oxidase structures in H_2_O (r.m.s.d. 0.23 Å to PDB-ID 3L9G [9]). No major differences between the structures at ambient temperature and 100 K were observed.

**Figure 4:**
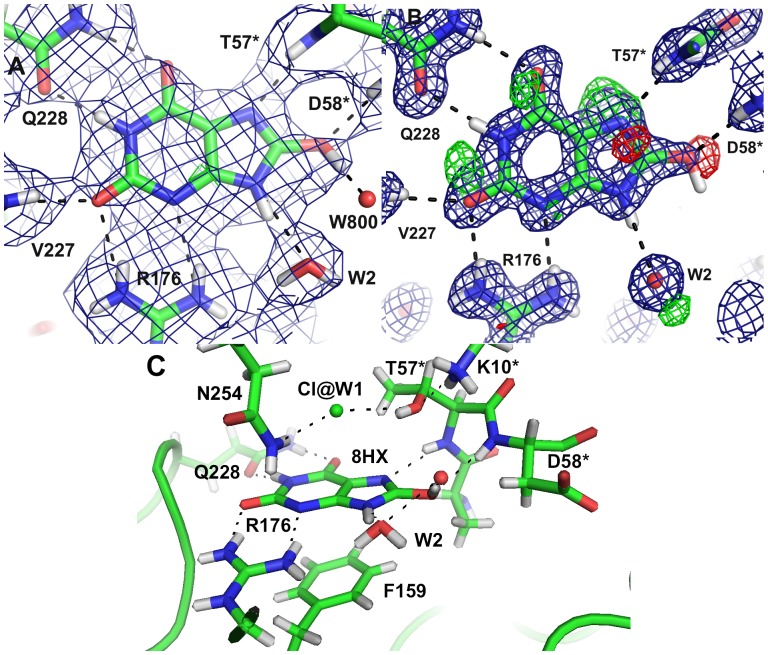
The active site with the substrate 8-hydroxyxanthine A) in the neutron structure B) in the 1.06 Å X-ray structure showing the 2mF_o_–DF_c_ map contoured at 1.5 σ (blue) and the mF_o_–DF_c_ map contoured at 3.0 σ (positive green, negative red); C) An overview of the catalytic site.

For the crystals grown in the presence of uric acid, the 1.06 Å X-ray structure showed that an intact urate backbone is present at ∼80% occupancy. The identity of the species contributing the remaining 20% is not evident; there are positive mF_o_–DF_c_ peaks near N7 below the purine plane (Fig. 4B), between O2 and N1 and near O6 above the purine plane. The O8 is clearly visible in the 2mF_c_–DF_c_ map, but there is a 4.4 σ negative mF_o_–DF_c_ peak, consistent with the 80% occupancy.

In the initial mF_o_–DF_c_ nuclear-scattering density map of the active site, deuterium atoms were seen on the 8-hydroxyxanthine N9 and O8 atoms. It was surprising that N1 appeared to be unprotonated, which seemed improbable based on electronic structure calculations [30]. Modelling a deuterium on N1 did not produce significant negative peaks in the mF_o_–DF_c_ map (Figure 4A), so we chose this chemically more reasonable model. The 2.9 Å distance between N1 of 8-hydroxyxanthine and Oε1 of Q228 suggests that indeed there is a hydrogen bond and hence a proton, even though it is not visible in the initial mF_o_–DF_c_ map.

**Figure 5:**
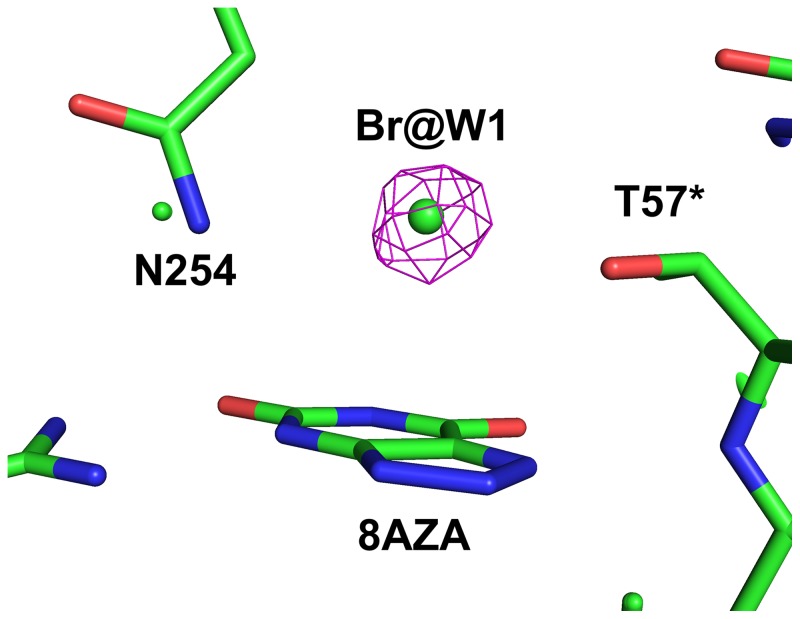
The anomalous difference Fourier map for the data collected at the Br absorption edge at 0.91994 Å contoured at 5.0 σ showing the W1 site occupied by Br^−^ and the inhibitor 8-azaxanthine in the active site.

The inhibitory effect of chloride allows us to observe the intact substrate in the active site [9]. Activities measured in the presence of 100 mM and 500 mM NaCl showed an inhibition of 22% and 65% respectively compared to the activity in the absence of NaCl. Refinement against the 1.06 Å X-ray data of this Cl^–^-inhibited structure clearly shows that the W1 site (the binding site of the co-substrate O_2_ or the nucleophilic water molecule) is occupied by a species heavier than water. To verify that a halide ion does bind to the W1-site, we replaced NaCl with NaBr in the crystallisation solution. Data were collected close to the Br absorption maximum at 0.91994 Å wavelength and the anomalous difference Fourier map showed a clear peak at the W1 site (Fig. 5), confirming the presence of a halide ion.

**Figure 6:**
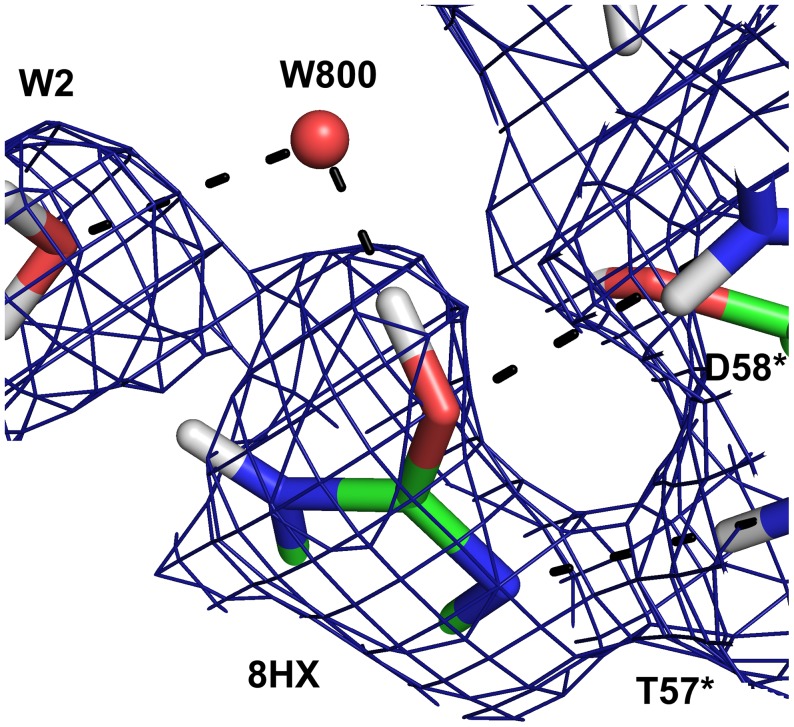
The surroundings of the hydroxyl group of 8-hydroxyxanthine in the neutron structure showing the 2mF_o_–DF_c_ OMIT map (blue) is contoured at 1.0 σ. The OMIT map was calculated omitting the 8-hydroxyxanthine molecule.

The neutron maps (Fig. 4A) reveal that the species in the active site is in fact not a urate anion in the keto tautomeric form, but 8-hydroxyxanthine monoanion N3, the enol tautomer that is less stable in water solution, as has been suggested by theoretical calculations [10]. The nuclear scattering density OMIT maps calculated without including the substrate (Fig. 6) clearly show the deuteron on O8, hydrogen bonded to a water molecule (W800).

**Figure 7:**
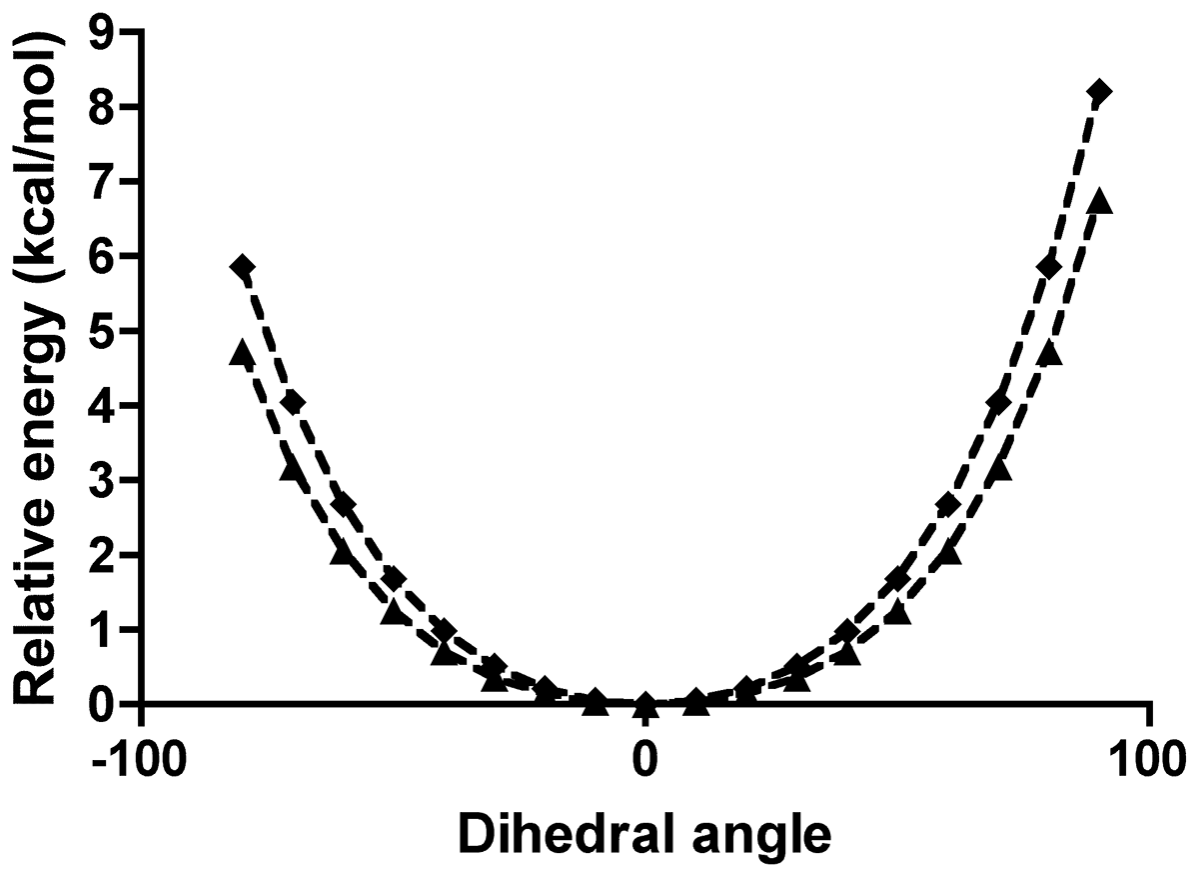
The relative electronic energy of uric acid (squares) and its monoanion N3 (triangles) as a function of the angle between the N7–H7 vector and the plane of the five-ring.

In solution the N3 monoanion of 8-hydroxyxanthine is calculated to be 70 kJ/mol less stable than the keto tautomer, but in the active site the order is reversed and 8-hydroxyxanthine is 55 kJ/mol more stable owing to the favourable interactions with back-bone amide group of T57* and W800. The pyramidalisation of N7 is energetically feasible both in neutral uric acid and in the monoanion (Fig. 7): The N7–H7 bond may deviate from the backbone plane by 50° at a cost of less than 2 kcal/mol thus preventing any collision. The keto tautomer urate could still bind in the active site despite the steric clash between H7 and the main-chain amide proton of T57*, but it would be rapidly tautomerised to 8-hydroxyxanthine.

**Figure 8:**
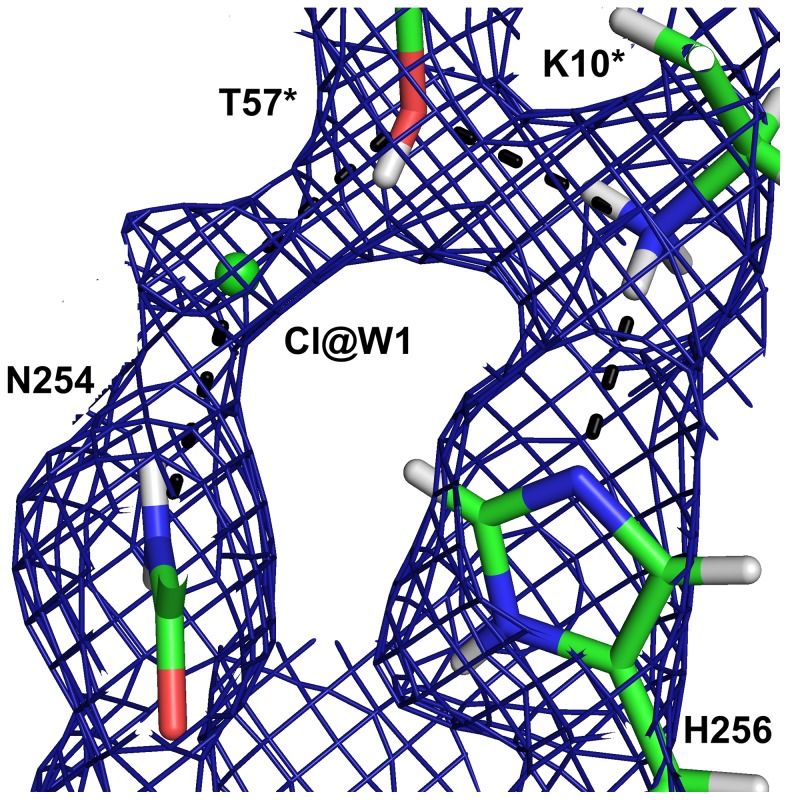
The catalytic triad in the neutron structure with 8-hydroxyxanthine. The neutron 2mF_o_–DF_c_ map (blue) is contoured at 1.0 σ.

The protonation state of the postulated catalytic triad formed by T57*, K10* and H256 is depicted in Fig. 8. Hγ of T57* forms a hydrogen bond with the Cl^−^ at the W1 site and the oxygen of this threonine accepts a hydrogen bond from K10*.

For H256, protons are observed on both Nδ and Nε. As a doubly protonated histidine is an unlikely species at pD 8.5, especially as the Nε atom forms a hydrogen bond to the amine group of K10*, the density can be explained either by a triplet state with a protonated neutral histidinyl radical–8-hydroxyxanthine radical anion pair, or a singlet state with 50% occupancy of each hydrogen. Consequently K10* is only 50% deprotonated in the singlet state. To distinguish between these models that give identical nuclear scattering density maps, we performed QM/MM calculations with a doubly protonated H256 and deprotonated K10* in both the singlet and triplet states. The singlet state is 300 kJ/mol more stable than the triplet state in the QM/MM calculations, which strongly suggests that the crystal structure is a singlet state. This implies that the effect of the chloride ion is to prevent the initial deprotonation that allows a one-electron transfer to an intermediate acceptor.

**Figure 9:**
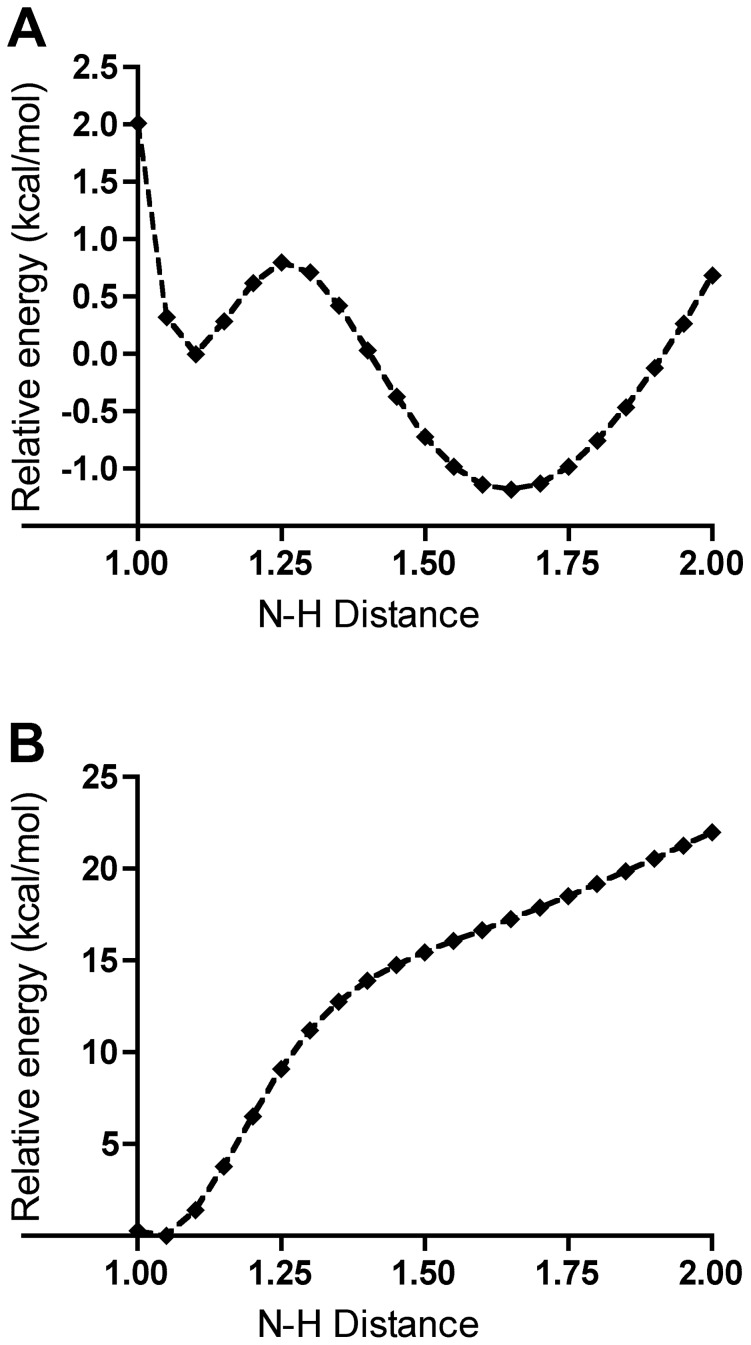
The calculated relative electronic energies of the 5-methylimidazole–proton–aminomethane model system as a function of the methylimidazole-N3–H distance for the positively charged singlet (A) and neutral doublet (B) states.

An isolated model system consisting of a 5-methylimidazole protonated on N1 mimicking H256, a proton and aminomethane mimicking K10* was studied by QM. Plotting the electronic energy of the system against the distance between the proton and the amine nitrogen (Fig. 9) shows two minima, with the proton preferably being localised on the Lys model, in accordance with the relative p*K*_a_ values of the two residues. This model illustrates the possible existence of two stable positions for the proton with a low energy barrier between them and a very fast interconversion rate. This result is consistent with our crystallographic data: the nuclear scattering density maps calculated from both configurations are essentially identical and the occupancy of the deuterons on H256 refines to 51% and 49% and the occupancy of the lysine Hζ3 to 53%. This implies that when H256 Nε2 is protonated and Nδ1 is deprotonated, K10* has to be deprotonated.

To summarise, in the singlet state QM/MM model, the H256 Hε2 atom consistently moves to K10* as expected. On the other hand, in the triplet state, H256 remains protonated and the spin density is localised mainly on the histidine and the urate radical anion. This result is also in accordance with a QM evaluation made on the doubly protonated 5-methylimidazole–proton–aminomethane model, differing from the previous one by being a neutral doublet. The proton–nitrogen distance scan shows that the proton is firmly bound to the imidazole (Fig. 9B).

In order to reproduce the crystal structure in the QM geometry optimisation we added a water molecule to the vicinity of O8. This water molecule forms a hydrogen bond with the hydroxyl proton of 8-hydroxyxanthine. In the anaerobic structure [13], a water molecule is also observed at this site. On the other hand in our 1.06 Å resolution X-ray structure there is a ∼3σ peak in the mF_o_–DF_c_ map at this position, but modelling a water molecule produces a high negative peak in the mF_o_–DF_c_ map. This suggests that in our structures, this water molecule is disordered.

**Figure 10:**
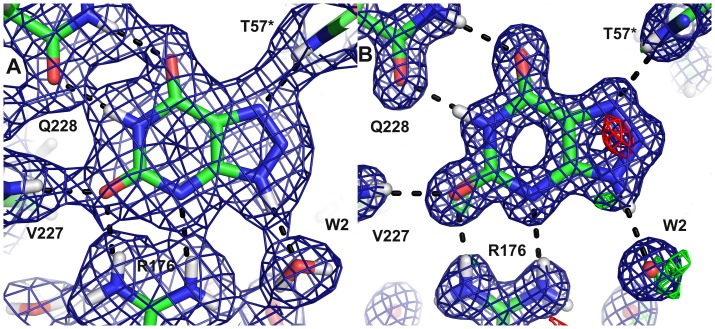
The active site with the inhibitor 8-azaxanthine in the neutron structure showing the 2mF_o_–DF_c_ map contoured at 1.0 σ (blue) and the mF_o_–DF_c_ map contoured at 3.0 σ (positive green, negative red). B) The corresponding maps in the 1.1 Å X-ray structure contoured at 1.5 σ and 3.0 σ, respectively.

**Figure 11:**
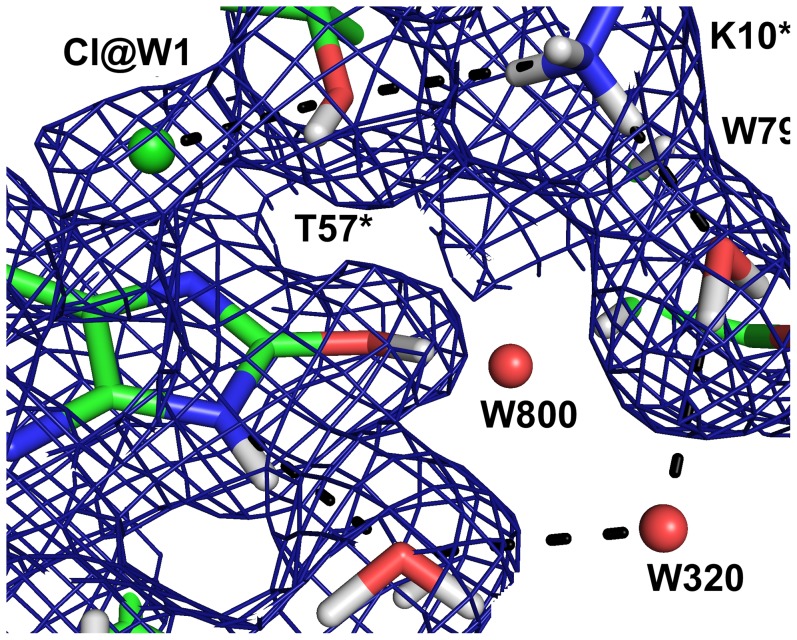
The proposed proton relay system in the neutron structure with 8-hydroxyxanthine showing the 2mF_o_–DF_c_ map contoured at 1.0 σ.

**Figure 12:**
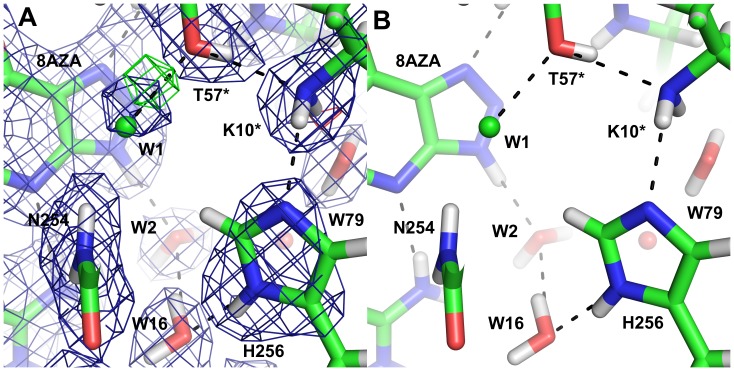
The catalytic triad in the neutron structure with 8-azaxanthine. A) The neutron 2mF_o_–DF_c_ map (blue) is contoured at 1.5 σ and the mF_o_–DF_c_ map (green) at 3 σ. B) The hydrogen-bonding network between W2 and H256.

In the complex with the inhibitor 8-azaxanthine, which cannot react due to its high ionisation energy, the species in the active site was the N3 monoanion of 8-azaxanthine (Fig. 10), analogous to the 8-hydroxyxanthine monoanion described above. The hydrogen bonding within the active site is somewhat different for 8-azaxanthine than for 8-hydroxyxanthine. The water molecule W800 that is mobilised by the 8-hydroxyl group is not present in the 8-azaxanthine structure. This water molecule is essential in the mechanism, transmitting the 8-OH proton to the proton-relay system (Fig. 11). In the structure with 8-azaxanthine the deuteron exchange between K10* and H256 is no longer present (Fig. 12) with the deuteron localised on K10*. It appears that there are multiple configurations of the deuteron arrangement between K10*, T57* and the chloride ion. Besides the configuration shown in Fig. 12 the positive mF_o_–DF_c_-peak suggests that a configuration where, like in the 8-hydroxyxanthine complex, the T57* Hγ points towards the chloride also exists. The resolution of the neutron data is not sufficient to refine both conformations.

A chain of hydrogen bonds connects N9 of the substrate to the W1 site through the conserved water molecule W2, waters W320 and W79 and the catalytic triad K10*–T57*–H256 (Fig. 11). As N9 is deprotonated to generate the intermediate dehydrourate, this proton-relay system allows the concomitant protonation of the peroxide species in the W1 site. The nuclear scattering length density along this chain is diffuse, indicating that protons are being shuffled back and forth.

## Conclusions

The chloride anion traps the N3 monoanion 8-hydroxyxanthine before any electron transfers occur. This means that the inhibitory effect of chloride is not only based on replacing the molecular oxygen, but it also blocks the deprotonation of the substrate that leads to electron transfer to a protein acceptor even in the absence of oxygen [13], apparently by strongly decreasing the second acid dissociation constant of the substrate. As this deprotonation is concomitant with a one-electron transfer from the resulting dianion, this explains why the chloride anion blocks the reaction at such an early stage. Our structure is also very similar to the recent urate oxidase structure determined under anaerobic conditions [13] (r.m.s.d. 0.11 Å).

**Figure 13:**
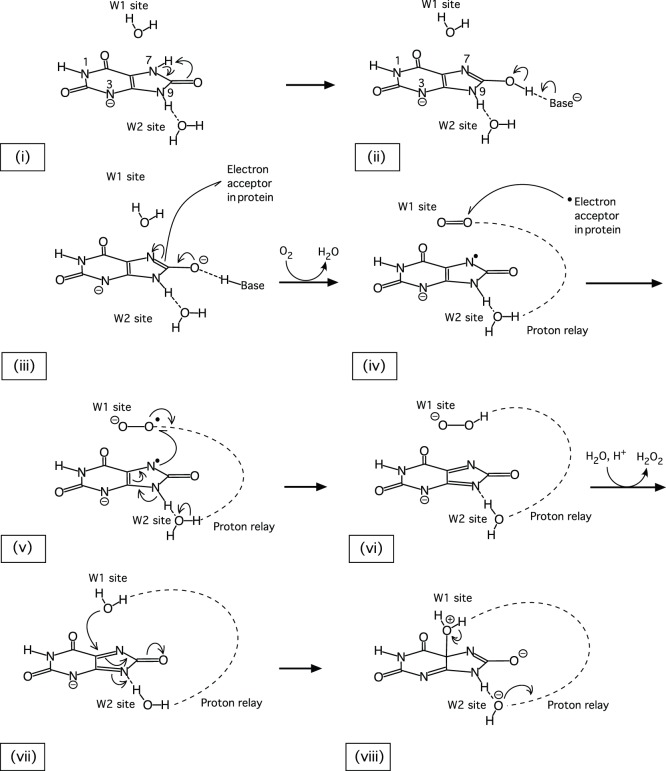
The postulated first steps of the reaction mechanism.

We have obtained two neutron structures of urate oxidase, which provide unique information on the proton positions and allow us to propose a consistent mechanism (Fig. 13) for the crucial initial stages of the reaction. The urate N3 monoanion binds in the active site ((i), Fig. 13) and rapidly tautomerises to form 8-hydroxyxanthine ((ii), Fig. 13). The deprotonation of 8-hydroxyxanthine ((iii), Fig. 13) is concomitant with an electron transfer to an intermediate acceptor in the protein, creating a triplet state. When molecular oxygen occupies the W1 site ((iv), Fig. 13), it can accept the electron, followed by the unpaired electron from the urate radical anion ((v), Fig. 13), which leads to the dehydrourate intermediate. After protonation through the proton relay chain ((vi), Fig. 13), the hydroperoxide is replaced by the nucleophilic water ((vii), Fig. 13), which, activated by the proton-relay system, attacks the dehydrourate and generates 5-hydroxyisourate.

The neutron structures of urate oxidase together with high-resolution X-ray crystallography data and quantum chemical calculations have established unexpected tautomeric state of the substrate, thus solving a long-standing mechanistic conundrum concerning the site of deprotonation.
